# Origin and expansion of the mosquito *Aedes aegypti* in Madeira Island (Portugal)

**DOI:** 10.1038/s41598-018-38373-x

**Published:** 2019-02-19

**Authors:** Gonçalo Seixas, Patrícia Salgueiro, Aline Bronzato-Badial, Ysabel Gonçalves, Matias Reyes-Lugo, Vasco Gordicho, Paulo Ribolla, Bela Viveiros, Ana Clara Silva, João Pinto, Carla A. Sousa

**Affiliations:** 1 Global Health and Tropical Medicine, Instituto de Higiene e Medicina Tropical, Universidade Nova de Lisboa, Lisboa, Portugal; 2 Departamento de Parasitologia, Instituto de Biociências, Universidade Estadual Paulista, Botucatu, São Paulo Brazil; 3 Museum of Natural History of Funchal, RAM, Funchal, Madeira Portugal; 4 Sección Entomologia Médica, Instituto de Medicina Tropical, Universidad Central de Venezuela, Caracas, Venezuela; 5 Departamento de Saúde, Planeamento e Administração Geral, Instituto de Administração da Saúde e Assuntos Sociais, IP-RAM, Funchal, Madeira Portugal; 6 Present Address: Vice-Presidency of Madeira Regional Government, Advisor For Health and Social Affairs, Funchal, Madeira Portugal

## Abstract

Historically known as the yellow fever mosquito, *Aedes aegypti* invaded Madeira Island in 2005 and was the vector of the island’s first dengue outbreak in 2012. We have studied genetic variation at 16 microsatellites and two mitochondrial DNA genes in temporal samples of Madeira Island, in order to assess the origin of the invasion and the population structure of this mosquito vector. Our results indicated at least two independent colonization events occurred on the island, both having a South American source population. In both scenarios, Venezuela was the most probable origin of these introductions, a result that is in accordance with the socioeconomic relations between this country and Madeira Island. Once introduced, *Ae. aegypti* has rapidly expanded along the southern coast of the island and reached a maximum effective population size (*N*_*e*_) in 2012, coincident with the dengue epidemic. After the outbreak, there was a 10-fold reduction in *N*_*e*_ estimates, possibly reflecting the impact of community-based vector control measures implemented during the outbreak. These findings have implications for mosquito surveillance not only for Madeira Island, but also for other European regions where *Aedes* mosquitoes are expanding.

## Introduction

Arbovirus transmission is becoming an increasing public health threat in Europe, mainly due to the establishment of invasive mosquito vectors and importation of arboviruses by viremic travelers^1^. The Asian tiger mosquito, *Aedes albopictus*, was first recorded in the European continent in Albania, in 1979^2^. Since then, this mosquito invaded most of central and western Europe and become established in 27 countries^3^. Coincidently, epidemics of chikungunya and dengue have been reported over the last 20 years, notably in Italy (2007, 2017)^4,5^, France (2010, 2017)^6,7^ and Croatia (2010)^8^. Another mosquito species responsible for arbovirus transmission is *Aedes aegypti*, previously present in Europe until mid-20^th^ century and re-established in Madeira and in the Black Sea region^3^.

**Figure 1:**
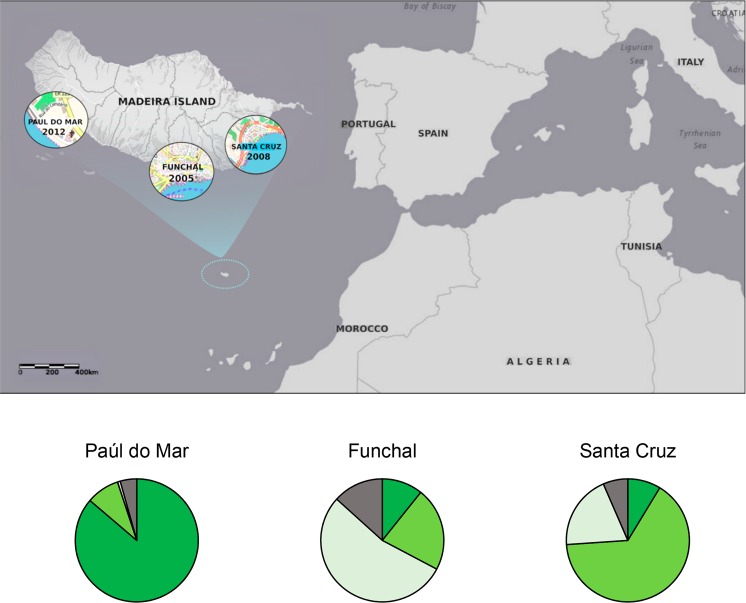
Madeira Island map showing sampling sites: Pául do Mar, a fishing village in the western point of *Ae. aegypti* distribution in the island; Funchal, the capital, where the main harbour is present; Santa Cruz, near the only Airport of the island. Below each locality name is the year of the first report of the introduction of the species. Pie charts indicate proportions of individuals assigned (Tq = 0.50) to each of the three genetic clusters determined by STRUCTURE (See text). Grey colour indicate admixed individuals with no cluster assignment. The map was produced using ArcGIS 10.2 (Esri, Redlands, CA).

In the Portuguese island of Madeira, *Ae. aegypti* was first reported in 2005, in the vicinity of Funchal city. Since then, this mosquito has subsequently expanded its distribution throughout the southern coast of the island^9,10^, being detected in Santa Cruz (East) in 2008 and in Paúl do Mar (West) in 2012 (Fig. 1). The presence of this mosquito in the island, coupled with the introduction of DENV-1, led to an outbreak of dengue fever, with more than 2000 notified cases between October 2012 and March 2013^11^. The epidemic led to the reinforcement of vector control activities in the island for the subsequent months, particularly in the more densely populated area of Funchal city^12^. Implemented anti-vector activities included larval control through massive salt application in the city’s storm drains and community educational campaigns in order to remove flower dishes, the main *Ae. aegypti* breeding site in Madeira^13^. Targeted insecticide/biocide application was performed in health facilities and one school located in the most affected Funchal area, using pyrethroids and *Bacillus thurigiensis israelensis* (Bti)^12^.

Madeira is a famous touristic destination, mostly for Europeans, with daily flights from/to several European countries and regular stops of cruise ships^12,14^. This scenario increases the risk of exportation of both *Ae. aegypti* and viremic individuals to Europe^12^. In fact, the dengue epidemic in Madeira was responsible for 78 imported cases that were notified in 13 different countries, the majority corresponding to tourists that had travelled to the island during the outbreak^15^. To mitigate the risk of importation/exportation of virus and vectors, local health authorities perform vector control activities at the International Airport and at the Funchal harbour, and coordinate an island-wide integrated Madeira Dengue Surveillance System (MDSS), responsible for the detection of imported cases.

Despite its insular condition, Madeira has a considerable risk of importing exotic vectors and pathogens from tropical regions. This is mainly due to the strong socio-economic relations that the island maintains with South American countries, such as Brazil and Venezuela^14^. Coincidently, phylogenetic analysis and an importation index based on the air-travel interconnectivity with dengue-endemic countries revealed Venezuela as the most likely country of origin for the circulating DENV-1 in the island^16^. In addition, previous studies showed that *Ae. aegypti* from Madeira is able to transmit dengue, chikungunya and Zika viruses^17,18^, pinpointing the potential risk of local arbovirus transmission.

Previous genetic analyses involving different markers such as mitochondrial DNA, knockdown resistance associated genes^19^, microsatellites^20^, and Single Nucleotide Polymorphisms^21^, provided evidence for a South American origin of the introduced *Ae. aegypti* population in Madeira. However, these analyses did not have sufficient resolution to precisely pinpoint the geographic origin and colonization dynamics of the *Ae. aegypti* Madeira population, mainly because (i) a single sample from the island was used; (ii) some of the most important putative source populations were not included. Moreover, these studies did not provide information about the dynamics of the colonization of this mosquito in the island.

In this study, we analyzed microsatellites and mtDNA genes in *Ae. aegypti* samples from different localities of Madeira island collected at different time-points, as well as from additional sites in South America. In addition, genetic data were integrated with global genetic data available for this species in order to address the following questions:

1. What is the population genetic structure and demographic history of *Ae. aegypti* in Madeira island?
2. Are these populations the result of a single or multiple mosquito introductions?
3. What is the most likely country of origin of the source populations from which *Ae. aegypti* was introduced?

## Results

### Microsatellite genetic variation

Forty-eight out of 246 (19.5%) exact tests of Hardy-Weinberg proportions were significant (Supplementary Table S3). The majority of these departures were associated with positive *F*_*is*_ values, indicative of heterozygote deficits. Most heterozygote deficits were detected at a single locus, AC4, which accounted for 15 out of the 48 significant tests. Micro-checker results suggested that locus AC4 had a high probability of having null alleles in all but Fx05L and PM14A samples (Supplementary Table S3). The consistent heterozygote deficits and suspicion of null alleles lead us to remove locus AC4 from subsequent analyses of population structure. There were a total of 348 significant pairwise genotypic association tests out of 1851 performed. However, no pair of loci was consistently associated across samples, which suggests an absence of linkage disequilibrium among loci.

Microsatellite polymorphism in *Ae. aegypti* from Madeira was low to moderate, with mean over sample AR ranging from 1.7 (AC7) to 6.6 (88AT1) and mean *He* from 0.081 (AC7) to 0.789 (88AT1) (Supplementary Table S3). Expected heterozygosity was significantly lower in the other two localities of Madeira when compared to the mean over-years of Funchal (Wilcoxon signed-rank tests, Paúl do Mar: *p* = 0.001; Santa Cruz: *p* = 0.004). In Paúl do Mar, no significant differences in *He* were found between 2013 and 2014 (Wilcoxon signed-rank test, *p* = 0.168). The proportion of unrelated individuals obtained by ML-RELATE was above 70% in all samples except for Santa Cruz. This sample had the highest frequency of related individuals (41%) with a high proportion of full sibs (15%) and backcrosses (20%).

The overall mean AR and *He* across temporal samples from Madeira was significantly different from those of the Brazilian samples (Wilcoxon signed-ranks tests, Santos: AR *p* < 0.001, *He p* = 0.033; São Sebastião: AR *p* = 0.018, *He p* = 0.015) but comparable to the sample of Caracas, Venezuela (Wilcoxon signed-ranks tests, Caracas: AR *p* = 0.159, *He p* = 0.433).

With the exception of Funchal in 2014, when the larval sample (Fx2014L) was less polymorphic, estimates of *He* and AR were largely similar between larval and adult samples collected in the same locality and year (Supplementary Table S3). The degree of relatedness among individuals was comparable between larval and adult samples, as shown in Supplementary Fig. S1. These results suggest that the genetic variation captured by both sampling methods is comparable. Therefore, larval and adult samples were pooled in subsequent temporal analyses to represent a single sample per collection year.

### Effective population size and demographic stability

In Funchal, single-sample estimates of effective population size based on the linkage disequilibrium method (LD-*N*_*e*_) increased overtime, from a minimum of 3.4 in 2005 to a maximum of 657.0 in 2012, the year of the dengue epidemic (Table 2). After 2012, there was ten-fold reduction of LD-*N*_*e*_. This pattern of temporal variation was not evident in the two-sample estimates of *N*_*e*_ (*F*_*s*_-*N*_*e*_, Table 2). These estimates varied between 291.1 and 401.3 with no apparent trend for increase/decrease over years and with overlapping 95%CI.

For both methods, *N*_*e*_ estimates were consistently higher in Funchal, when compared to the other localities (Table 2). In Paúl do Mar, there was a 5-fold increase of LD-*N*_*e*_ between 2013 and 2014. The lowest LD-*N*_*e*_ estimate was obtained for the only sample available from Santa Cruz. The estimates of LD-*N*_*e*_ obtained for the three South American continental samples analyzed in this study were consistently lower than those obtained for Funchal and Paúl do Mar, except for the samples of 2005 and 2013, respectively (Table 2).

Significant departures from mutation-drift equilibrium were detected by heterozygote tests only under the TPM model (Table 2). There was a consistent surplus of loci with apparent heterozygote excess in all Madeiran samples, but these were significant only in 2012 and onwards. In Funchal, the sample of 2014 also showed a shifted allele frequency distribution, indicative of a recent bottleneck. In continental samples, heterozygosity tests suggest a recent bottleneck in the population of São Sebastião, Brazil. The sample of Caracas, Venezuela, presented a shifted allele frequency distribution but the corresponding heterozygosity test was only marginally significant (*p* < 0.05).

### Population structure and origin

**Figure 2:**
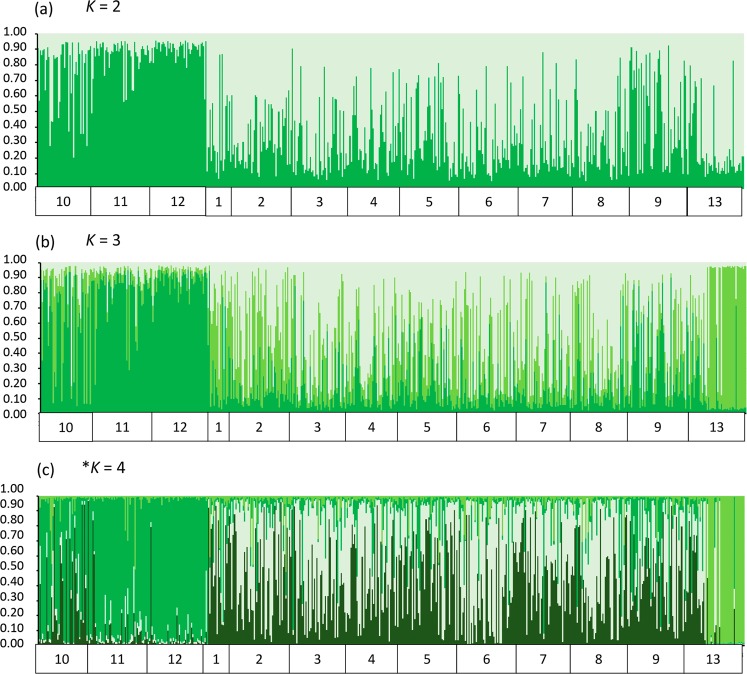
Genetic structure of Madeira *Ae. aegypti* populations using 15 microsatellite markers. Each bar represents an individual with the colour of the bar giving the probability of the individual belonging to a genetic population or cluster. (**a,b,c**) STRUCTURE plots of Madeira populations with *K* number of clusters as indicated. An asterisk indicates the plot representing the optimal *K* as determined by the delta *K* method. Legend: 1–9: Funchal populations; 10–12: Paúl do Mar populations; 13- Santa Cruz population. For additional population details, see Table 1.

**Figure 3:**
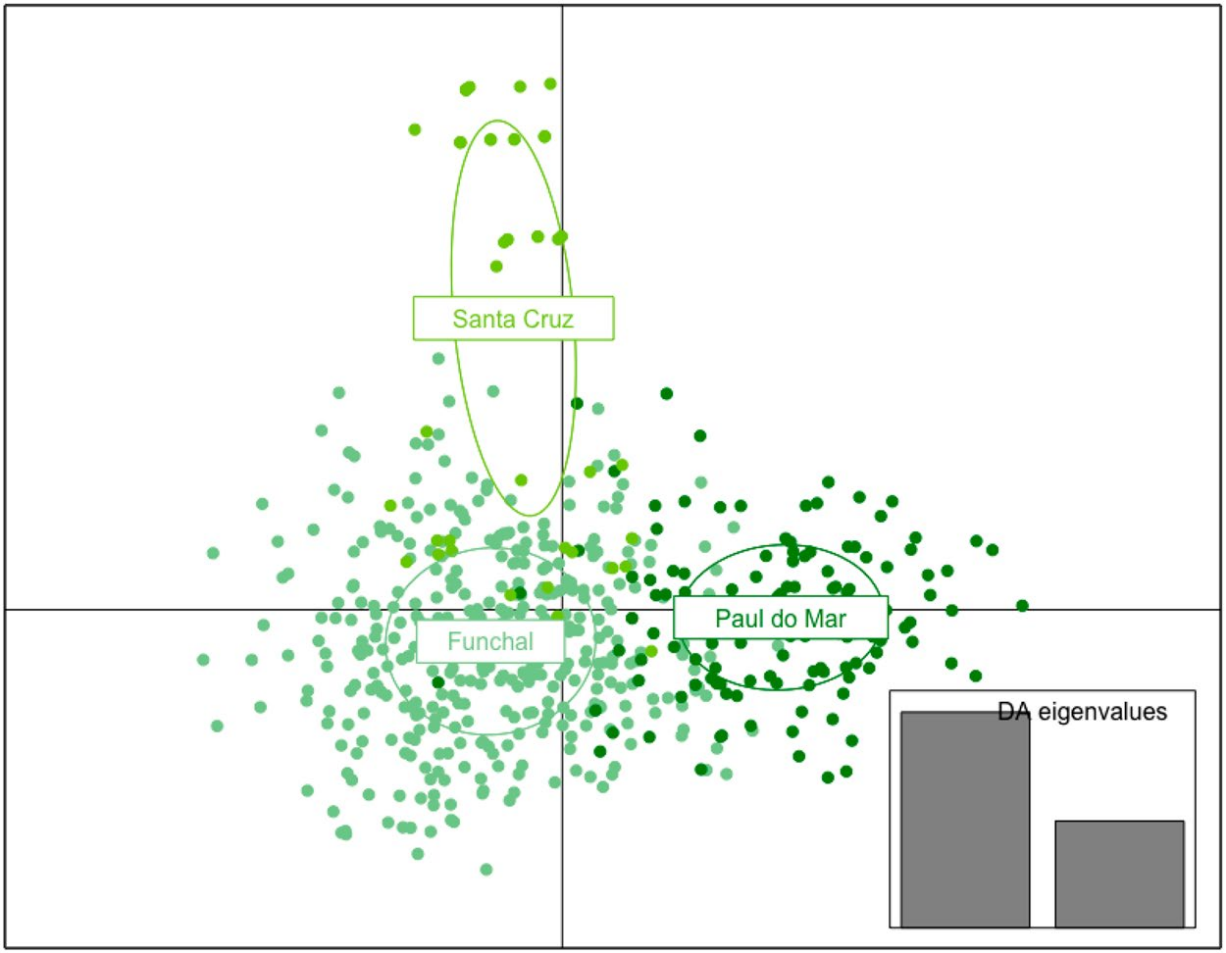
Discriminant analysis of principal components (DAPC) of *Ae. aegypti* populations in Madeira. Same populations depicted in the STRUCTURE plot shown in Fig. 2.

A first STRUCTURE analysis was performed with the Madeira dataset only and the results for the three best *K* values (*K* = 2 to *K* = 4) are shown in Fig. 2. Graphical representations of Evanno’s *ΔK* can be seen in Supplementary Fig. S2. The sample from Paúl do Mar consistently formed a homogenous distinct genetic cluster in the three population structure scenarios. In the *K* = 3 clusters scenario, population partitioning corresponds to the geographic localities sampled, with distinct genetic clusters for Santa Cruz, Paúl do Mar and Funchal. The *K* = 4 scenario maintains the geographic substructuring but separates the samples from Funchal into two different genetic backgrounds. This genetic partitioning within Funchal was not confirmed by the DAPC analysis (Fig. 3). Madeira samples were divided into two principal genetic clusters. The first discriminant function separates Paúl do Mar from Funchal and Santa Cruz while subdivision of these two localities in discriminant function two is less pronounced, judging from the respective eigenvalues.

**Figure 4:**
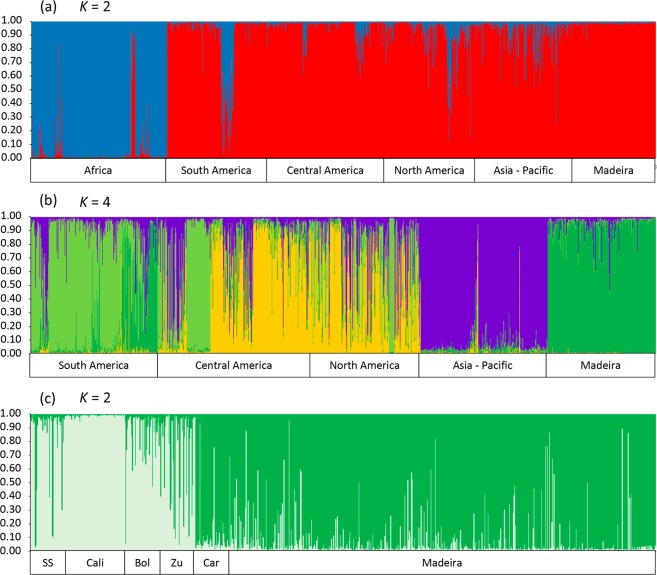
Analyses of *Ae. aegypti* from Madeira using 11 microsatellite markers. (**a**) STRUCTURE plot separating Aaa = *Ae. ae. aegypti*, red cluster; Aaf = *Ae. ae. formosus*, blue cluster. (**b**) Genetic structure of pantropical *Ae. aegypti* populations. (**c**) Genetic relationships between Madeira and South American populations. Colors of (**a,b**) are presented as in Gloria-Soria *et al*.^20^. Legend: SS – São Sebastião, Brazil; Cali – Cali, Colombia; Bol – Bolivar, Venezuela; Zu – Zulia, Venezuela; Car – Caracas, Venezuela.

A second STRUCTURE analysis was conducted with the complete dataset comprising the samples of Madeira and South America genotyped in this study, along with the dataset of Gloria-Soria *et al*.^20^. The best *K* obtained was *K* = 2, reflecting the known segregation of the African *Aedes aegypti formosus* from out-of-Africa *Aedes aegypti aegypti* populations (Fig. 4a; see also Gloria-Soria *et al*.^20^). All Madeiran individuals were homogenously assigned to the *Ae. aegypti aegypti* group. When the analysis was repeated without the African samples, the best value of *K* was equal to four (Fig. 4b). This partitioning reflected the previously shown three continental Asian/Pacific, North-Central American and South American clusters^20^ along with one additional cluster that grouped all Madeira island samples with a subset of South-American samples. A third STRUCTURE analysis was performed with samples of the fourth cluster only (Fig. 4c). This analysis gave a best *K* = 2 and grouped the Madeiran samples mainly with Venezuelan samples from Caracas. A few individuals from São Sebastião, Brazil, were also assigned to this Madeira/Caracas cluster. The other cluster comprised individuals mainly from Brazil, Colombia and non-Caracas Venezuelan samples.

**Figure 5:**
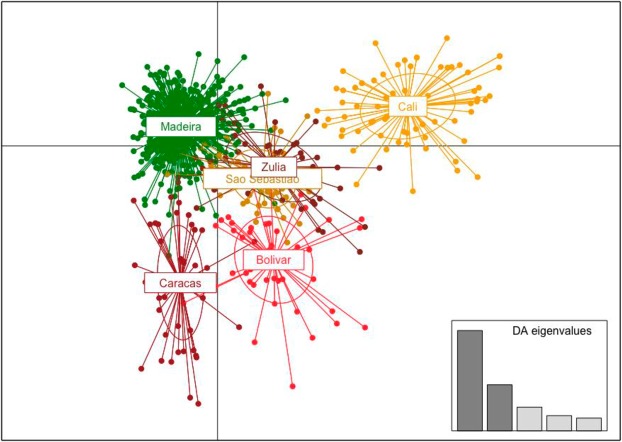
Discriminant analysis of principal components (DAPC) of *Ae. aegypti* populations using a Madeira/South America subset. Same populations depicted in the STRUCTURE plot shown in Fig. 4c.

Results of a DAPC analysis conducted with the Madeira/South America subset confirmed a closer relationship between Madeira Island and the samples of Caracas, Venezuela, and São Sebastião, Brazil (Fig. 5).

### Mitochondrial DNA analysis

Summary statistics of genetic variation for each mtDNA gene in Madeira Island are shown in Supplementary Table S4. Partial COI sequences were obtained for 202 individuals (Supplementary Table S6). The 764 bp alignment revealed the presence of three distinct haplotypes and nucleotide diversity (*π*) of 0.00317. Partial ND4 sequences were analyzed from 191 mosquitoes (Supplementary Table S5). The 351 bp alignment revealed the presence of four haplotypes and *π* = 0.00545. Neutrality tests were non-significant for both genes.

**Figure 6:**
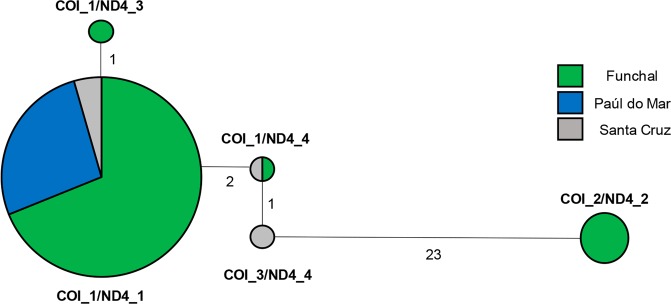
Median-joining network based on haplotypes obtained from the mtDNA concatenated COI and ND4 sequences as generated by Network version 5. The size of the nodes corresponds to the number of individuals with corresponding haplotypes. The number indicates the number of mutations between each haplotype.

A Median-Joining haplotype network for the concatenated COI/ND4 sequences is shown in Fig. 6. Of the five different haplotypes identified, haplotype COI_1/ND4_1 was present in over 90% of all individuals in all localities and it was the only haplotype detected in Paúl do Mar in the two years sampled (Table 3). The second most frequent haplotype (COI_2/ND4_2) was separated by 23 mutational steps from the central COI_1/ND4_1 and it was only observed in Funchal. The two most frequent haplotypes were consistently detected in Funchal since the first collection in 2005. Three additional low frequency haplotypes derived from COI_1/ND4_1 by one or two mutational steps. One of these, COI_3/ND4_4 was unique to Santa Cruz only (2014) and haplotype COI_1/ND4_3 was unique to the 2005 collection of Funchal, in a single individual. Haplotype COI_1/ND4_4 was detected in 2014 simultaneously in Funchal and Santa Cruz. The 21 mtDNA sequences obtained from samples of Caracas, Venezuela, were all of the same haplotype, COI_1/ND4_1.

**Figure 7:**
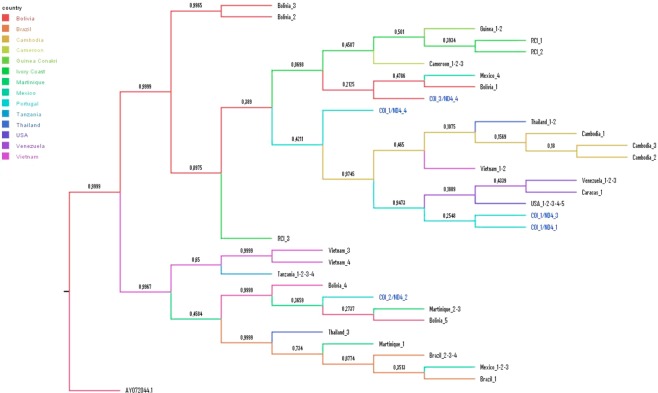
Phylogenetic tree obtained with a Bayesian inference of concatenated COI and ND4 sequences. Sequence number AY072044.1 is an outgroup *Aedes albopictus* specimen.

We performed a phylogenetic analysis with concatenated COI-ND4 sequences to infer the phylogenetic relationships between Madeira and worldwide *Ae. aegypti* sequences^22^. The resulting phylogenetic tree revealed that *Ae. aegypti* in Madeira is represented by members of the two major mtDNA lineages known for this species (Fig. 7)^23^. The main COI_1/ND4_1 is included in the West African lineage and clusters with Venezuelan and USA haplotypes. Haplotypes COI_1/ND4_3, COI_1/ND4_4 and COI_3/ND4_4 are also included in this clade. Haplotype COI_2/ND4_2 is included in the East Africa lineage that contains sequences from Asia, Central America, Caribe and Brazil.

## Discussion

The results of the present study indicate that the recently established *Ae. aegypti* population of Madeira has derived from at least two independent introductions, possibly occurring at different time-points. Venezuela is the most likely geographic origin but a Brazilian source population, at least for one of the introductions, cannot be fully excluded. After the initial colonization, *Ae. aegypti* has rapidly expanded throughout the southern part of the island, reaching its maximum effective population size in 2012, which was coincident with the dengue outbreak that occurred in Madeira^12^. The reduction of *N*_*e*_ recorded after the outbreak may have been the result of the increased vector control implemented to halt virus transmission.

The lower mtDNA genetic diversity of *Ae. aegypti* in Madeira is consistent with a recently established island population. Values of haplotype diversity below 0.200 recorded in the island are lower than those recorded for continental populations of this mosquito species (e.g. Brazil^24^: *Hd* = 0.800; Florida, USA^25^: *Hd* = 0.886; Colombia^26^: *Hd* = 0.914). However, microsatellite genetic diversity of this 2005 sample (Fx05L) is comparable to other *Ae. aegypti* island^27^ and mainland populations^28,29^. Furthermore, MDE tests did not provide evidence of a population contraction associated with a founder event mediated by only a few individuals. This may reflect a higher evolution rate of microsatellites, with mutation rates^30^ around 10^−4^–10^−3^, or that the size of the founding population was still sufficient to maintain a representative gene pool of this species.

Both genetic and historical evidence support an initial introduction of *Ae. aegypti* in Funchal, possibly by maritime transportation. Funchal is the capital and the major urban centre of the island, where the international harbour is located. It was in Funchal that the first *Ae. aegypti* specimens were collected in October 2005^9^. In agreement, Funchal displays the highest genetic diversity for both mtDNA and microsatellites and the largest *N*_*e*_ estimates recorded in Madeira, suggesting a longer established population. After the introduction, *Ae. aegypti* rapidly expanded eastwards and westwards of Funchal, reaching Santa Cruz in 2008^10^ and Paúl do Mar in 2012^12^. Again, genetic data supports an earlier colonization of Santa Cruz, judging from the higher haplotype diversity when compared to Paúl do Mar, with a single haplotype. The lower *N*_*e*_ estimates also agree with subsequent colonisations of those two localities after the initial introduction of *Ae. aegypti* in Funchal. This expansion was most probably human-mediated, through road transportation along the only highway that connects most of the southern part of the island. In fact, the pronounced island’s topography is likely to act as a natural barrier to active dispersal of this mosquito and this may be the reason why *Ae. aegypti* has not yet established in the northern part of the island. The influence of human movement in shaping genetic diversity, structure and differentiation was also observed on other *Ae. aegypti* insular populations as in the Antilles islands^31^ and in the Pacific region^32^.

Concatenated mtDNA sequences revealed the presence of two highly divergent haplotypes separated by 23 mutation steps (COI_1/ND4_1 and COI_2/ND4_2). These two haplotypes were detected in the initial sample of Funchal 2005, providing evidence that the initial colonization was made by at least two different maternal lineages. However, the presence of a unique mtDNA haplotype (COI_3/ND4_4) in Santa Cruz may suggest one additional introduction on the island. Santa Cruz is a county where the International Airport of Madeira is located, providing an opportunity for airplane-mediated transportation of mosquitoes, an occurrence that has been previously detected^33^. While this haplotype was only detected in 2014, we cannot exclude an earlier introduction due to the lack of sample availability from previous years in this locality.

Both Bayesian clustering and DAPC analyses suggest a South American origin of *Ae. aegypti* in Madeira. All Madeira samples grouped in a distinct genetic cluster together with specimens from Venezuela, mainly from Caracas. Moreover, the most frequent mtDNA haplotype in Madeira (COI_1/ND4_1) is the same haplotype found in all Caracas specimens sequenced in this study. A Venezuelan origin of *Ae. aegypti* in Madeira is not surprising given that Madeira has an important emigrant community living in Caracas^34^. During summer, there is extensive movement between Caracas and Madeira because of holidaying by the migrant community. Coincidently, the only direct flight connecting Madeira and South America is between Funchal and Caracas^16^.

A Venezuelan origin of *Ae. aegypti* also agrees with the insecticide susceptibility profile of *Ae. aegypti* in Madeira. This population was found to be resistant to three different insecticide classes and resistance was associated with knockdown resistance (*kdr*) mutations F1534C and V1016I and elevated expression of detoxification enzymes^35^. Similarly, insecticide resistance studies in *Ae. aegypti* from Venezuela revealed high frequencies of F1534C and V1016I *kdr* mutations and increased activity of glutathione-S transferases, esterases and mixed-function oxidases^36,37^, the same profile as that observed in Madeiran *Ae. aegypti*.

In addition to a Venezuelan origin, at least one introduction may have derived from Brazil. There were 7 individuals from São Sebastião, Brazil, which grouped in the Madeira/Caracas genetic cluster and 21 individuals from Madeira with genetic ancestry closest to the Brazilian/Colombian cluster. Moreover, the phylogenetic tree indicates that haplotype COI_2/ND4_2 groups in a clade in sequences from Brazil but not from Venezuela. However, this may simply reflect that the number of specimens sequenced from Venezuela was too low to capture all the haplotype diversity. Therefore, we cannot exclude the possibility of haplotype COI_2/ND4_2 also being present in Venezuela but not sampled.

Estimates of LD*-N*_*e*_ in Funchal consistently increased overtime from the first sample time-point in 2005 until reaching its highest value in 2012. Coincidently, 2012 was the year of the dengue epidemic on the island^38^. Such an increase in *N*_*e*_ agrees with the rapid expansion of this mosquito vector on the island. The precise ecological conditions driving this expansion are not fully understood but the mild temperate climate suitable for sustaining a mosquito population throughout the year coupled with an extensive availability of breeding sites (flower pots)^13^ in urban and rural areas may have facilitated adaptation and subsequent population expansion on the island^14^.

The utility of genetic markers in assessing the impact of vector control on the mosquito population has been tested previously, with varying degrees of success^39–41^. Interestingly, estimates of *F*_*s*_*-N*_*e*_ did not show any trend of temporal variation. While two-sample estimates are sensitive to population fluctuations, these methods are influenced by the initial (*T*_0_) genetic variation of the population, since *N*_*e*_ is retrieved from an unbiased estimate of allelic variance^42,43^. Therefore, the initial low microsatellite polymorphism of the Madeiran *Ae. aegypti* population may have affected the sensitivity of *F*_*s*_*-N*_*e*_ in detecting population contractions. Coincidently, *F*_*s*_*-N*_*e*_ samples of Funchal in the order of the hundreds are within the average values obtained in a previous study analyzing a global dataset for *Ae. aegypti*^44^.

After the 2012 outbreak, estimates of *N*_*e*_ significantly decreased in 2013 and 2014. Heterozygosity tests and mode-shift allele detected a recent bottleneck during this period. These results suggest that the vector control measures implemented after the dengue outbreak were effective in reducing *Ae. aegypti* densities. It should be emphasized that vector control during the dengue outbreak of Madeira was predominantly based on community-based larval source reduction, enforced by a strong communication campaign led by the local health authorities^12^. Given the high insecticide resistance of *Ae. aegypti* on the island, alternative non-insecticidal methods are essential to contain the mosquito population and, subsequently, prevent arbovirus transmission. In this context, larval source reduction is a valid option for Madeira. This method is also recommended by the World Health Organization as a primary vector control tool for *Ae. aegypti*^45^.

To conclude, *Ae. aegypti* has recently arrived in Madeira and rapidly expanded its population size to levels able to sustain transmission of an arbovirus epidemic. The Venezuelan origin is coherent with the socioeconomic relations of this insular territory with that country and highlights the importance of monitoring mosquito populations at points of entry such as international harbours and airports. This study also provided evidence for the effectiveness of non-insecticidal vector control methods. The relatively small effective size of this island vector population may also be regarded as advantageous for the implementation of vector control tools that rely on genetically modified mosquitoes^46^.

## Methods

### Mosquito samples

Mosquito collections were performed in three localities of Madeira: Funchal, the capital city; Santa Cruz and Paúl do Mar, representing the eastern and western distribution limits of *Ae. aegypti* in the island (Fig. 1). Collections were made at six time points in Funchal (2005, 2009, 2011, 2012, 2013 and 2014) and two (2013 and 2014) in Paúl do Mar. In addition, mosquito samples from two localities in Brazil (Santos and São Sebastião) and one in Venezuela (Caracas) were also analysed. The two Brazilian localities represent coastal cities with major international harbours. Caracas is the capital of Venezuela and located ca. 30 km away from the major harbour city La Guaira. Sample details, including the sampling method, collection year and sample sizes, are available in Table 1.

**Table 1:** *Aedes aegypti* samples included in this study.

Sample code^a^	Sample name	Country	Locality	GPS coordinates	Collection method	Collection month/year	Sample size
1	Fx05L	Portugal	Funchal	32.64134,−16.916687	Larval	November/2005	22
2	Fx09L	Portugal	Funchal		Larval	October/2009	48
3	Fx11A	Portugal	Funchal		BG-traps	October/ 2011	45
4	Fx12A	Portugal	Funchal		BG-traps	October/2012	46
5	Fx12L	Portugal	Funchal		Ovitrap	October/2012	46
6	Fx13A	Portugal	Funchal		BG-traps	October/2013	46
7	Fx13L	Portugal	Funchal		Ovitrap	October/2013	46
8	Fx14A	Portugal	Funchal		BG-traps	October/2014	46
9	Fx14L	Portugal	Funchal		Ovitrap	October/2014	46
10	PM13L	Portugal	Paúl do Mar	32.75907, −17.230439	Ovitrap	October/2013	46
11	PM14A	Portugal	Paúl do Mar		BG-traps	October/2014	46
12	PM14L	Portugal	Paúl do Mar		Ovitrap	October/2014	46
13	SC14L	Portugal	Santa Cruz	32.689282, −16.79074	Ovitrap	October/2014	46
—	Car	Venezuela	Caracas	10.480594, −66.903606	Ovitrap	April/2013	46
—	ST	Brazil	Santos	−23.967882, −46.328887	Ovitrap	NA/2008	47
—	SS	Brazil	São Sebastião	−23.806347, −45.401653	Ovitrap	NA/2008	47
—	Gloria-Soria^20^	NA	NA	NA	NA	NA	3566

NA, not applicable; ^a^sample codes used in Fig. 2.

**Table 2:** Estimates of effective population size and Mutation-Drift Equilibrium tests.

	Mutation-drift equilibrium tests				Effective population size		
Population	Year	SMM	TPM	Mode-Shift	LD-*N*_*e*_	Year interval	*F*_*s*_ - *N*_*e*_
Funchal	2005	10 (0.299)	11 (0.027)	Normal	3.4 [2.3–8.7]		
	2009	9 (0.244	10 (0.009)	Normal	42.9 [28.0–75.0]	2005–2009	304.9 [185.1–454.3]
	2011	9 (0.339)	11 (0.015)	Normal	71.2 [38.5–222.4]	2009–2011	401.3 [240.1–603.3]
	2012	9 (0.165)	12 **(0.001)**	Normal	657.0 [166.9–∞]	2011–2012	291.1 [175.4–435.7]
	2013	10 (0.103)	12 **(<0.001)**	Normal	54.9 [40.2–78.6]	2012–2013	383.1 [230.8–573.3]
	2014	11 (0.047)	12 **(<0.001)**	Shifted	49.9 [36.5–71.1]	2013–2014	295.2 [176.6–443.8]
Paúl do Mar	2013	6 (0.227)	11 **(<0.001)**	Normal	6.0 [3.6–9.2]		
	2014	8 (0.178)	9 (0.017)	Normal	30.6 [21.8–44.1]	2013–2014	62.2 [34.8–97.4]
Santa Cruz	2014	7 (0.596)	9 (0.312)	Normal	1.2 [1.0–1.4]		
Caracas	2013	10 (0.116)	10 (0.021)	Shifted	23.9 [11.1–68.8]		
Santos	2008	6 (0.939)	10 (0.138)	Normal	10.8 [8.7–13.2]		
São Sebastião	2008	9 (0.380)	12 **(<0.001)**	Normal	12.1 [8.5–17.2]		

MDE tests: 95% confidence intervals in square brackets. Upper values, number of loci (out of 15) in which *H*_*e*_ > *H*_*eq*_; lower values are the *p-value* for the corresponding one-tailed Wilcoxon test; SMM: stepwise-mutation model; TPM (30%): two-phased model with 30% of indels greater than one repeat; In bold: significant tests after adjustment by the sequential Bonferroni procedure; Effective population size: generations sampled in the two-sample estimates were set at 0 and 10 based on the length of time between the two field collections.

**Table 3:** Haplotype frequencies for concatenated COI/ND4 genes from *Ae. aegypti* populations in Madeira.

		N	COI_1/ND4_1	COI_1/ND4_3	COI_1/ND4_4	COI_2/ND4_2	COI_3/ND4_4
Funchal	2005	14	0.86	0.07	0.00	0.07	0.00
Funchal	2009	16	1.00	0.00	0.00	0.00	0.00
Funchal	2011	9	0.56	0.00	0.00	0.44	0.00
Funchal	2012	28	0.89	0.00	0.00	0.11	0.00
Funchal	2013	32	0.88	0.00	0.00	0.12	0.00
Funchal	2014	27	0.89	0.00	0.04	0.07	0.00
Paúl do Mar	2013	15	1.00	0.00	0.00	0.00	0.00
Paúl do Mar	2014	28	1.00	0.00	0.00	0.00	0.00
Santa Cruz	2014	9	0.78	0.00	0.11	0.00	0.11
	Total Madeira	178	0.90	0.006	0.01	0.08	0.006

Both immature and adult mosquitoes were sampled. Collected immatures (eggs and larvae) were reared to adults under insectary conditions. Adults were identified to species using morphological keys^47^ and stored individually in silica-gel at −20 °C until DNA extraction.

### DNA extraction

Genomic DNA was extracted using the NZY tissue gDNA isolation kit (NZYtech Portugal) for the 2005 Madeira sample, and with the protocol of Collins *et al*.^48^ for the other Madeira samples. DNA of the individuals from Brazil and Venezuela were extracted using a Chelex100® Molecular Biology Grade resin (Bio-rad Laboratories) protocol according to the manufacturer’s protocols.

### Microsatellite genotyping

A total of 16 microsatellites were genotyped by fragment size analysis of polymerase chain reaction amplified products. Primer sequences and PCR conditions followed previously published protocols^49–51^ and are described in Supplementary Table S1. Fragment size analysis was performed by capillary electrophoresis on an ABI3130xl genetic analyser (Applied Biosystems), at the DNA Analysis Facility at Science Hill, Yale University. Microsatellite alleles were scored using GENEMARKER software (SoftGenetics, PA, USA).

The genotypes obtained were integrated into the microsatellite genotypic database of Gloria-Soria *et al*.^20^, available in VectorBase (Project ID: VBP0000138). This database has genotypes for 12 of the 16 microsatellites genotyped in a total of 3,566 individuals from 78 countries representing Asian, African and American *Ae. aegypti* populations. In order to calibrate inter-lab allele scoring, 20 individuals from Gloria-Soria *et al*.^20^, kindly provided by the Jeffrey Powell laboratory at Yale University, were analysed at GHTM and the genotypes were compared with the original scorings.

The genotypes obtained for the samples here analysed are available in VectorBase (Project ID: VBP0000303).

### Microsatellite data analysis

Expected heterozygosity (*H*_*e*_) and the inbreeding coefficient (*F*_*is*_) were estimated using GENEPOP^52^. The same software was used to perform exact tests of departure from Hardy-Weinberg proportions and of linkage disequilibrium (LD) among pairs of loci. Estimates of allele richness (AR) were obtained for each population by the statistical rarefaction approach implemented in HP-RARE^53^. The software Micro-checker 2.2.3^54^ was used to test for the presence of null alleles (99% confidence interval) at each locus/sample.

Two estimates of current effective population size (*N*_*e*_) were made: single-sample estimates based on the linkage disequilibrium method^55^; and two-sample temporal estimates, based on the *F*-statistic of Jorde & Ryman^56^. Calculations were performed using NeEstimator v2^57^. Because rare alleles may bias linkage disequilibrium *N*_*e*_ estimates, alleles with frequency below 0.05 at each locus were removed from the analysis.

Evidence of recent population perturbations was assessed by heterozygosity tests as implemented in BOTTLENECK version 1.2.02^58^. Expected heterozygosity estimates assuming mutation drift equilibrium (MDE) were calculated from the number of alleles and sample size under two mutation models, considered more suitable for microsatellites: the stepwise mutation model (SMM) and a two-phased model (TPM) with 30% multistep mutations (variance = 30%). Although the SMM has been considered as better suited for the type of mutation process most frequent in microsatellites (i.e. DNA slippage^59^), there is evidence that intermediate mutation models such as the TPM with increasing proportions of multistep mutations are less prone to detect false positives^60^. Wilcoxon tests were used to assess significance between observed and MDE-expected heterozygosities, as recommended for analysis with less than 20 loci^61^. In addition, the allele frequency distribution method was also used^60^. Under MDE, an L-shaped allele frequency distribution is expected, whereas a shifted distribution due to loss of low-frequency alleles is consistent with a recent bottleneck^62^.

In order to assess the degree of relatedness among individuals, the maximum-likelihood method implemented in ML-RELATE was used^63^. For each pair of individuals, log-likelihood estimates are calculated for four pedigree classes: unrelated, half-siblings, full-siblings and parent-offspring.

Bayesian clustering analysis was performed using the software STRUCTURE version 2.3.4.^64^, in order to assess within-island population subdivision and to determine the most likely source populations of *Ae. aegypti* in Madeira. In a first analysis, only Madeira island samples were used. Subsequently, the Madeira island genotypes were analysed with the continental sample dataset of Gloria-Soria *et al*.^20^. Twenty independent runs were made for each value of *K*, which varied from one to 10 for within island analysis, including source population determination, and from one to five at the subspecies/species level. Each run was conducted with a burn-in of 100,000 iterations and 500,000 replicates, assuming an admixture model with correlated allele frequencies. The optimal number of clusters was determined following the guidelines of Pritchard *et al*.^64^ and the delta *K* statistic of Evanno *et al*.^65^, using STRUCTURE HARVESTER version 0.6.94^66^. The information from the outputs of each *K* was aligned by the Greedy method implemented in CLUMPP^67^.

Discriminant Analysis of Principal Components (DAPC), as implemented by ADEGENET^68^, was used to visualize patterns of genetic differentiation among individual mosquitoes belonging to different genetic clusters in a two-dimensional plot. This analysis was performed with the samples from Madeira and a subset of candidate source populations selected from the Bayesian clustering analysis.

Whenever multiple tests were performed, the nominal significance level (α = 0.05) was adjusted by the sequential Bonferroni procedure^69^.

### Mitochondrial DNA sequencing

The mitochondrial genes COI and ND4 were analysed by direct sequencing from amplified products, corresponding to 764 bp and 351 bp, respectively, using previously published primers^22,24^ and protocols^19^. In addition to Madeira individuals, mtDNA sequences for 21 individuals from Caracas, Venezuela, were also obtained to compensate for the scarcity of sequences available in Genbank for this country. Sequences were aligned and manually corrected using BioEdit v7.0.5^70^. For each gene, haplotype diversity (*Hd*), nucleotide diversity (π) and the Tajima and Fu and Li neutrality tests were computed by DNAsp v5.10^71^.

In order to infer the relationships between haplotypes in Madeira, haplotype networks were constructed for concatenated COI-ND4 sequences using a median-joining algorithm as implemented in the NETWORK software^72^.

The BEAST v1.8.4 software^73^ was used to generate a phylogenetic tree based on the COI-ND4 concatenated haplotypes, obtained from sequences produced in this study and others retrieved from GenBank (Supplementary Table S2). The analysis was run in three separate independent runs with 500 million generations, sampled every 100 000 runs for the concatenated genes. A Bayesian skyline population growth model was used. The substitution model HKY^74^ with gamma and invariant sites and three partitions into codon positions was selected. MCMC analysis was run long enough for convergence to be obtained. To analyze convergence and stability, we used Tracer v1.6 software^75^. TreeAnnotator was used to estimate the final Maximum Clade Credibility Tree, summarizing the posterior probability of each clade of the trees, as well as the average and confidence interval for the evolutionary rate of each branch of the tree. The obtained Bayesian trees were visualized and edited with FIGTREE 1.4.3. (http://tree.bio.ed.ac.uk/software/figtree/).

## Supplementary information

Supplementary Information

Table S3

## References

[1] Fernandez-Garcia MD (2016). Chikungunya virus infections among travellers returning to Spain, 2008 to 2014. Euro Surveill.

[2] Adhami J, Murati N (1987). The presence of the mosquito *Aedes albopictus* in Albania. Rev Mjekësore.

[3] Medlock JM (2012). A Review of the Invasive Mosquitoes in Europe: Ecology, Public Health Risks, and Control Options. Vector-Borne Zoonotic Dis.

[4] Rezza G (2007). Infection with chikungunya virus in Italy: an outbreak in a temperate region. Lancet.

[5] Venturi G (2017). Detection of a chikungunya outbreak in Central Italy Detection of a chikungunya outbreak in Central. Euro Surveill.

[6] Gould EA, Gallian P, De Lamballerie X, Charrel RN (2010). First cases of autochthonous dengue fever and chikungunya fever in France: from bad dream to reality!. Clin Microbiol Infect.

[7] Calba C (2017). Preliminary report of an autochthonous chikungunya outbreak in France, July to September 2017. Euro Surveill.

[8] Gjenero-Margan, I. *et al*. Autochthonous dengue fever in Croatia, August-September 2010. *Euro Surveill* **16** (2011). 21392489

[9] Margarita Y (2006). First record of *Aedes* (Stegomyia) *aegypti* (Linnaeus, 1762) (Diptera, Culicidae) in Madeira Island – Portugal (Portuguese, English abstract). Acta Parasitol Port.

[10] Gonçalves Y (2008). On the presence of *Aedes* (Stegomyia) *aegypti* Linnaeus, 1762 (Insecta, Diptera, Culicidae) in the island of Madeira (Portugal). Bol Mus Mun Funchal.

[11] Alves MJ (2013). Clinical presentation and laboratory findings for the first autochthonous cases of dengue fever in Madeira island, Portugal, October 2012. Euro Surveill.

[12] European Centre for Disease Prevention and Control. Dengue outbreak in Madeira, Portugal, March 2013. Stockholm: ECDC; (2014).

[13] Nazareth T (2014). Strengthening the perception-assessment tools for dengue prevention: a cross-sectional survey in a temperate region (Madeira, Portugal). BMC Public Health.

[14] Lourenço, J. & Recker, M. The 2012 Madeira dengue outbreak: epidemiological determinants and future epidemic potential. *PLoS Negl Trop Dis* **8** (2014). 10.1371/journal.pntd.0003083 PMC4140668 25144749

[15] Frank C, Höhle M, Stark K, Lawrence J (2013). More reasons to dread rain on vacation? Dengue fever in 42 German and United Kingdom Madeira tourists during autumn 2012. Euro Surveill.

[16] Wilder-Smith A (2014). The 2012 dengue outbreak in Madeira: exploring the origins. Euro Surveill.

[17] Seixas G (2018). Potential of Aedes aegypti populations in Madeira Island to transmit dengue and chikungunya viruses. Parasit. Vectors.

[18] Jupille H, Seixas G, Mousson L, Sousa CA, Failloux AB (2016). Zika Virus, a New Threat forEurope?. PLoS Negl. Trop. Dis..

[19] Seixas, G. *et al*. *Aedes aegypti* on Madeira Island (Portugal): genetic variation of a recently introduced dengue vector. *Mem Inst Oswaldo Cruz* 1–8, 10.1590/0074-0276130386 (2013). 10.1590/0074-0276130386 PMC4109174 24473797

[20] Gloria-Soria A (2016). Global genetic diversity of *Aedes aegypti*. Mol Ecol.

[21] Gloria-Soria A (2018). Origin of a high-latitude population of *Aedes aegypti* in Washington, DC. Am J Trop Med Hyg.

[22] Paupy, C. *et al*. Genetic structure and phylogeography of *Aedes aegypti*, the dengue and yellow-fever mosquito vector in Bolivia. *Infect Genet Evol*, 10.1016/j.meegid.2012.04.012 (2012). 10.1016/j.meegid.2012.04.012 22522103

[23] Bracco JE, Capurro ML, Lourenço-de-Oliveira R, Sallum MAM (2007). Genetic variability of *Aedes aegypti* in the Americas using a mitochondrial gene: evidence of multiple introductions. Mem. Inst. Oswaldo Cruz.

[24] Paduan KDS, Ribolla PEM (2008). Mitochondrial DNA polymorphism and heteroplasmy in populations of *Aedes aegypti* in Brazil. J Med Entomol.

[25] Damal K, Murrell EG, Juliano SA, Conn JE, Loew SS (2013). Phylogeography of *Aedes aegypti* (yellow fever mosquito) in South Florida: MtDNA evidence for human-aided dispersal. Am J Trop Med Hyg.

[26] Jaimes-Dueñez J, Arboleda S, Triana-Chávez O, Gómez-Palacio A (2015). Spatio-Temporal distribution of *Aedes aegypti* (Diptera: Culicidae) mitochondrial lineages in cities with distinct dengue incidence rates suggests complex population dynamics of the dengue vector in Colombia. PLoS Negl Trop Dis.

[27] Gloria-Soria, A. *et al*. Temporal genetic stability of *Stegomyia aegypti* (=*Aedes aegypti*) populations. *Med Vet Entomol*, 10.1111/mve.12153 (2016). 10.1111/mve.12153 PMC4856540 26744174

[28] Monteiro FA (2014). Genetic diversity of Brazilian *Aedes aegypti*: patterns following an eradication program. PLoS Negl Trop Dis.

[29] Kotsakiozi P (2017). Tracking the return of *Aedes aegypti* to Brazil, the major vector of the dengue, chikungunya and Zika viruses. PLoS Negl Trop Dis.

[30] Ellegren H (2000). Microsatellite mutations in the germline: Implications for evolutionary inference. Trends Genet.

[31] Sherpa S (2018). At the origin of a worldwide invasion: unraveling the genetic makeup of the Caribbean bridgehead populations of the dengue vector *Aedes aegypti*. Genome Biol. Evol..

[32] Calvez E (2016). Genetic diversity and phylogeny of *Aedes aegypti*, the main arbovirus vector in the Pacific. PLoS Negl. Trop. Dis..

[33] Ibañez-Justicia A (2017). The first detected airline introductions of yellow fever mosquitoes (*Aedes aegypti*) toEurope, at Schiphol International airport, the Netherlands. Parasit. Vectors.

[34] Dinneen M (2011). Portuguese immigrants in Caracas: social networks and transnational connectivity. Port J Soc Sci.

[35] Seixas G (2017). Insecticide resistance is mediated by multiple mechanisms in recently introduced *Aedes aegypti* from Madeira Island (Portugal). PLoS Negl Trop Dis.

[36] Alvarez LC, Ponce G, Oviedo M, Lopez B, Flores AE (2013). Resistance to malathion and deltamethrin in *Aedes aegypti* (Diptera: Culicidae) from western Venezuela. J Med Entomol.

[37] Alvarez LC, Ponce G, Saavedra-Rodriguez K, Lopez B, Flores AE (2015). Frequency of V1016I and F1534C mutations in the voltage-gated sodium channel gene in *Aedes aegypti* in Venezuela. Pest Manag Sci.

[38] Sousa CA (2012). Ongoing outbreak of dengue type 1 in the Autonomous Region of Madeira, Portugal: preliminary report. Euro Surveill.

[39] Pinto J (2002). Genetic structure of *Anopheles gambiae* (Diptera: Culicidae) in São Tomé and Príncipe (West Africa): implications for malaria control. Mol Ecol.

[40] Wondji C (2005). Impact of insecticide-treated bed nets implementation on the genetic structure of *Anopheles arabiensis* in an area of irrigated rice fields in the Sahelian region of Cameroon. Mol Ecol.

[41] Hodges TK (2013). Large fluctuations in the effective population size of the malaria mosquito *Anopheles gambiae* s.s. during vector control cycle. Evol Appl.

[42] Luikart G, Ryman N, Tallmon DA, Schwartz MK, Allendorf FW (2010). Estimation of census and effective population sizes: The increasing usefulness of DNA-based approaches. Conserv Genet.

[43] Wang J (2016). A comparison of single-sample estimators of effective population sizes from genetic marker data. Mol Ecol.

[44] Saarman NP (2017). Effective population sizes of a major vector of human diseases. Aedes aegypti. Evol Appl.

[45] WHO. Global strategy for dengue prevention and control 2012–2020, Geneve, Switzerland: World Health Organization (2012).

[46] Marsden CD (2013). An analysis of two island groups as potential sites for trials of transgenic mosquitoes for malaria control. Evol Appl.

[47] Ribeiro, H. & Ramos, H.C. Identification keys of the mosquitoes (Diptera: Culicidae) of Continental Portugal, Açores and Madeira. *European Mosquito Bulletin* **3**, pp.1–11 (1999).

[48] Collins FH (1987). A ribosomal RNA gene probe differentiates member species of the *Anopheles gambiae* complex. Am J Trop Med Hyg.

[49] Slotman MA (2007). Polymorphic microsatellite markers for studies of *Aedes aegypti* (Diptera: Culicidae), the vector of dengue and yellow fever. Mol Ecol Notes.

[50] Lovin DD (2009). Genome-based polymorphic microsatellite development and validation in the mosquito *Aedes aegypti* and application to population genetics in Haiti. BMC Genomics.

[51] Brown JE (2011). Worldwide patterns of genetic differentiation imply multiple ‘domestications’ of *Aedes aegypti*, a major vector of human diseases. Proc Biol Sci.

[52] Rousset F (2008). Genepop’007: a complete reimplementation of the Genepop software for Windows and Linux. Mol Ecol Resour.

[53] Kalinowski ST (2004). Counting alleles with rarefaction: private alleles and hierarchical sampling designs. Conserv Genet.

[54] Van Oosterhout C, Hutchinson WF, Wills DPM, Shipley P (2004). MICRO-CHECKER: software for identifying and correcting genotyping errors in microsatellite data. Mol Ecol Notes.

[55] Waples RS, Do C (2008). LDNE: A program for estimating effective population size from data on linkage disequilibrium. Mol Ecol Resour.

[56] Jorde PE, Ryman N (2007). Unbiased estimator for genetic drift and effective population size. Genetics.

[57] Do C (2014). NeEstimatorv2: Re-implementation of software for the estimation of contemporary effective population size (Ne) from genetic data. Mol Ecol Resour.

[58] Piry S, Luikart G, Cornuet JM (1999). Computer note. BOTTLENECK: a computer program for detecting recent reductions in the effective size using allele frequency data. J Hered.

[59] Slatkin M (1995). Hitchhiking and associative overdominance at a microsatellite locus. Mol Biol Evol.

[60] Peery MZ (2013). More precisely biased: Increasing the number of markers is not a silver bullet in genetic bottleneck testing. Mol Ecol.

[61] Cornuet JM, Luikart G (1996). Description and power analysis of two tests for detecting recent population bottlenecks from allele frequency data. Genetics.

[62] Luikart G, Allendorf FW, Cornuet JM, Sherwin WB (1998). Distortion of allele frequency distributions provides a test for recent population bottlenecks. J Hered.

[63] Kalinowski, S. T., Wagner, A. P. & Taper, M. L. ML-RELATE: a computer program for maximum likelihood estimation of relatedness and relationship. *Mol Ecol Notes* **6**, 10.1111/j.1471-8286.2006.01256.x (2006).

[64] Pritchard JK, Stephens M, Donnelly P (2000). Inference of population structure using multilocus genotype data. Genetics.

[65] Evanno G, Regnaut S, Goudet J (2005). Detecting the number of clusters of individuals using the software STRUCTURE: a simulation study. Mol Ecol.

[66] Earl DA (2012). & vonHoldt, B. M. Structure Harvester: A website and program for visualizing STRUCTURE output and implementing the Evanno method. Conserv Genet Resour.

[67] Jakobsson M, Rosenberg N (2007). CLUMPP: a cluster matching and permutation program for dealing with label switching and multimodality in analysis of population structure. Bioinformatics.

[68] Jombart T, Devillard S, Balloux F (2010). Discriminant analysis of principal components: A new method for the analysis of genetically structured populations. BMC Genet.

[69] Holm S (1979). A simple sequentially rejective multiple test procedure. Scandinavian Journal of Statistics.

[70] Hall TA (1999). BioEdit: a user-friendly biological sequence alignment editor and analysis program for Windows 95/98/NT. Nucleic Acids Symposium Series.

[71] Librado P, Rozas J (2009). DnaSPv5: A software for comprehensive analysis of DNA polymorphism data. Bioinformatics.

[72] Bandelt HJ, Forster P, Röhl A (1999). Median-Joining networks for inferring intraspecific phylogenies. Mol Biol Evol.

[73] Drummond AJ, Suchard MA, Xie D, Rambaut A (2012). Bayesian Phylogenetics with BEAUti and the BEAST 1.7. Mol Biol Evol.

[74] Hasegawa M, Kishino H, Yano TA (1985). Dating of the human-ape splitting by a molecular clock of mitochondrial DNA. J Mol Evol.

[75] Rambaut, A., Drummond, A. J., Xie, D., Baele, G. & Suchard, M. A. Posterior summarisation in Bayesian phylogenetics using Tracer 1.7. *Systematic Biology*. syy032, 10.1093/sysbio/syy032 (2018). 10.1093/sysbio/syy032 PMC6101584 29718447

