# Supplementary material for: Origin and expansion of the mosquito Aedes aegypti in Madeira Island (Portugal)
Source: Sci Rep. 2019 Feb 19;9:2241. doi: 10.1038/s41598-018-38373-x (PMC6381185; doi:10.1038/s41598-018-38373-x)

**Origin and expansion of the mosquito *Aedes aegypti* in Madeira Island (Portugal)**

# Authors:

Gonçalo Seixas^1^, Patrícia Salgueiro^1^, Aline Bronzato-Badial^2^, Ysabel Gonçalves^3^, Matias Reyes-Lugo^4^, Vasco Gordicho^1^, Paulo Ribolla^2^, Bela Viveiros^5^, Ana Clara Silva^5,^ ^†^, João Pinto^1^, Carla A. Sousa^1,^*

# Institutional affiliations:

1. Global Health and Tropical Medicine, Instituto de Higiene e Medicina Tropical, Universidade Nova de Lisboa, Lisboa, Portugal
2. Departamento de Parasitologia, Instituto de Biociências, Universidade Estadual Paulista, Botucatu, São Paulo, Brasil
3. Museum of Natural History of Funchal, RAM, Funchal, Madeira
4. Sección Entomologia Médica, Instituto de Medicina Tropical, Universidad Central de Venezuela, Caracas, Venezuela
5. Departamento de Saúde, Planeamento e Administração Geral, Instituto de Administração da Saúde e Assuntos Sociais, IP-RAM, Funchal, Madeira, Portugal. ^†^Present address: Vice-Presidency of Madeira Regional Government, Advisor For Health and Social Affairs, Funchal, Madeira, Portugal

# *Corresponding author

E-mail: [casousa@ihmt.unl.pt](mailto:casousa@ihmt.unl.pt)

**Supplementary Table S1 - Description of the 16 microsatellite markers used.**

| **Locus** |  | **Primer Sequence** | **Fluorescent Primer** | **Source** | **Analysis** |
| --- | --- | --- | --- | --- | --- |
| AC1 | For | TCCGGTGGGTTAAGGATAGA | M13-FAM | Slotman et al. (2007) | Within Madeira and worldwide analysis |
|  | Rev | ACTTCACGCTCCAGCAATCT |  |  |  |
| AC2 | For | AATACAACGCGATCGACTCC | M13-FAM | Slotman et al. (2007) |  |
|  | Rev | AACGATTAGCTGCTCCGAAA |  |  |  |
| AC4 | For | GCGAATCGGTTCCCATAGTA | M13-FAM | Slotman et al. (2007) |  |
|  | Rev | CTTTATCGATCGACGCCATT |  |  |  |
| AC5 | For | TGGATTGTTCTTAACAAACACGAT | M13-FAM | Slotman et al. (2007) |  |
|  | Rev | CGATCTCACTACGGGTTTCG |  |  |  |
| AG1 | For | AATCCCCACACAAACACACC | M13-HEX | Slotman et al. (2007) |  |
|  | Rev | GGCCGTGGTGTTACTCTCTC |  |  |  |
| AG2 | For | TCCCCTTTCAAACCTAATGG | M13-HEX | Slotman et al. (2007) |  |
|  | Rev | TTTGCCCTCGTATGCTCTCT |  |  |  |
| AG5 | For | TGATCTTGAGAAGGCATCCA | M13-HEX | Slotman et al. (2007) |  |
|  | Rev | CGTTATCCTTTCATCACTTGTTTG |  |  |  |
| CT2 | For | CGCAGTAGGCGATATTCGTT | M13-HEX | Slotman et al. (2007) |  |
|  | Rev | ACCACCACCAACACCATTCT |  |  |  |
| A1 | For | GACGTAAACCGAGTGGGAGA | M13-FAM | Brown et al. (2011) |  |
|  | Rev | GCATTTAACCGCGCTAGAAC |  |  |  |
| A9 | For | GCAGCATGCACTTCACATTT | M13-FAM | Brown et al. (2011) |  |
|  | Rev | CGAATGGCATCTGATTCAAG |  |  |  |
| B2 | For | GGAAACACTTGCAGGGACAT | M13-HEX | Brown et al. (2011) |  |
|  | Rev | GCAGATGGTGGCAGTAGTGA |  |  |  |
| B3 | For | GCAAGTTGCAAAGTGCTCAA | M13-HEX | Brown et al. (2011) |  |
|  | Rev | ACCCACCGTTTGCTTTGTAG |  |  |  |
| AG4 | For | AAAACCTGCGCAACAATCAT | M13-FAM | Slotman et al. (2007) | Within Madeira analysis |
|  | Rev | AAGGACTCCGTATAATCGCAAC |  |  |  |
| AC7 | For | TCGGCAAATTACCACAAACA | M13-FAM | Slotman et al. (2007) |  |
|  | Rev | CATTGGACTCGCTATAACACACA |  |  |  |
| 88AT1 | For | CGTCGACGTTATCTCCTTGTT | M13-HEX | Lovin et al. (2009) |  |
|  | Rev | CCAACGCAAGATGCAAGATA |  |  |  |
| 201AAT1 | For | GATCGTTCGACAGCATCTGA | M13-HEX | Lovin et al. (2009) |  |
|  | Rev | GGAAAGCTCATCGCCTACTG |  |  |  |

*All forward primers were designed with a short M13 tail at the start (TCCCAGTCACGACGT)

**Supplementary Table S2 - Geographic origin and GenBank accession number of sequences used in the phylogenetic analyses.**

| **COI** | **ND4** | **Name** |
| --- | --- | --- |
| JQ926682 | JQ926708 | Bolivia_1 |
| JQ926682 | JQ926707 | Bolivia_2 |
| JQ926683 | JQ926707 | Bolivia_3 |
| JQ926681 | JQ926705 | Bolivia_4 |
| JQ926676 | JQ926705 | Bolivia_5 |
| JQ926703 | JQ926718 | Brazil_1 |
| JQ926703 | JQ926719 | Brazil_2-3-4 |
| JQ926698 | JQ926713 | Mexico_1-2-3 |
| JQ926699 | JQ926714 | Mexico_4 |
| JQ926696 | JQ926711 | Martinique_1 |
| JQ926697 | JQ926712 | Martinique_2-3 |
| JQ926701 | JQ926726 | Venezuela_1-2-3 |
| JQ926684 | JQ926725 | USA_1-2-3-4-5 |
| JQ926691 | JQ926720 | Thailand_1-2 |
| JQ926692 | JQ926721 | Thailand_3 |
| JQ926685 | JQ926723 | Vietnam_1-2 |
| JQ926686 | JQ926724 | Vietnam_3 |
| JQ926687 | JQ926724 | Vietnam_4 |
| JQ926688 | JQ926722 | Cambodia_1 |
| JQ926689 | JQ926722 | Cambodia_2 |
| JQ926690 | JQ926722 | Cambodia_3 |
| JQ926704 | JQ926715 | Tanzania_1-2-3-4 |
| JQ926693 | JQ926709 | RCI_1 |
| JQ926694 | JQ926709 | RCI_2 |
| JQ926695 | JQ926710 | RCI_3 |
| JQ926700 | JQ926717 | Guinea_1-2 |
| JQ926702 | JQ926716 | Cameroon_1-2-3 |

**Supplementary Table S4**. **Summary statistics for mtDNA genes in *Ae. aegypti* from Madeira.**

|  | **N** | **S** | **H** | ***Hd*** | **π** | **D (Tajima)** | **D*** | **F*** | **Gene size** |
| --- | --- | --- | --- | --- | --- | --- | --- | --- | --- |
| **COI** | 202 | 14 | 3 | 0.198 | 0.00317 | 0.044 | 1.525 | 1.158 | 764 bp |
| **ND4** | 191 | 13 | 4 | 0.192 | 0.00545 | -0.353 | 0.822 | 0.458 | 351 bp |
| **COI/ND4** | 178 | 27 | 5 | 0.177 | 0.00345 | -0.5077 | 1.524 | 0.839 | 1115 bp |

N, sample size; S, number of segregating sites; H, number of haplotypes; Hd, haplotype diversity; π, nucleotide diversity; D, Tajima’s D statistic; D* and F*, Fu and Li’s statistics.

**Supplementary Table S5. MtDNA haplotype sequences for COI and ND4 across *Ae. aegypti* samples from Madeira Island.**

| **Haplotype** | **N** | **Polymorphic positions** | | | | | | | | | | | | |  |
| --- | --- | --- | --- | --- | --- | --- | --- | --- | --- | --- | --- | --- | --- | --- | --- |
| **ND4** |  | **0** | **0** | **0** | **1** | **1** | **1** | **1** | **2** | **2** | **2** | **2** | **2** | **3** |  |
|  |  | **1** | **2** | **7** | **3** | **4** | **5** | **9** | **1** | **3** | **6** | **7** | **7** | **1** |  |
|  |  | **6** | **2** | **3** | **3** | **2** | **5** | **0** | **7** | **5** | **8** | **1** | **7** | **3** |  |
| H1 | 171 | T | T | T | C | T | G | T | A | T | A | T | T | C |  |
| H2 | 16 | C | C | C | T | C | . | C | G | C | G | A | C | T |  |
| H3 | 1 | . | . | . | . | . | A | . | . | . | . | . | . | . |  |
| H4 | 3 | . | . | . | . | . | . | . | G | . | . | A | . | . |  |
|  | | | | | | | | | | | | | | | |
| **COI** |  |  |  |  |  |  |  |  |  |  |  |  |  |  |  |
|  |  | **0** | **0** | **0** | **0** | **1** | **1** | **1** | **3** | **3** | **4** | **4** | **4** | **4** | **6** |
|  |  | **0** | **6** | **7** | **7** | **5** | **6** | **8** | **1** | **3** | **1** | **6** | **6** | **9** | **8** |
|  |  | **1** | **7** | **0** | **9** | **1** | **3** | **4** | **3** | **4** | **2** | **3** | **9** | **3** | **2** |
| H1 | 180 | A | C | T | A | G | G | C | A | T | G | A | T | C | C |
| H2 | 19 | G | T | C | G | A | A | T | G | C | A | G | C | T | T |
| H3 | 2 | . | . | . | . | . | . | . | . | C | . | . | . | . | . |

N corresponds to the number of sample belonging to this haplotype.

**Supplementary Figure S1. Proportion (in percentage) of related and unrelated pairs of individuals as determined by ML-RELATE**. Legend: U - unrelated, HS - half-siblings, FS - full-siblings and PO - parent-offspring. NS refers to a non-significant difference between adult and larval samples using Chi-square test (*p* > 0.05).

**Supplementary Figure S2.** **Graphics of Evanno’s *ΔK* for the different Bayesian clustering analysis implemented by STRUCTURE.** (a) All Madeira samples (Figure 2), (b) Worldwide dataset and Madeira (Figure 4a), (c) *Ae. aegypti aegypti* and Madeira samples (Figure 4b), (d) Madeira and South America samples (Figure 4c).


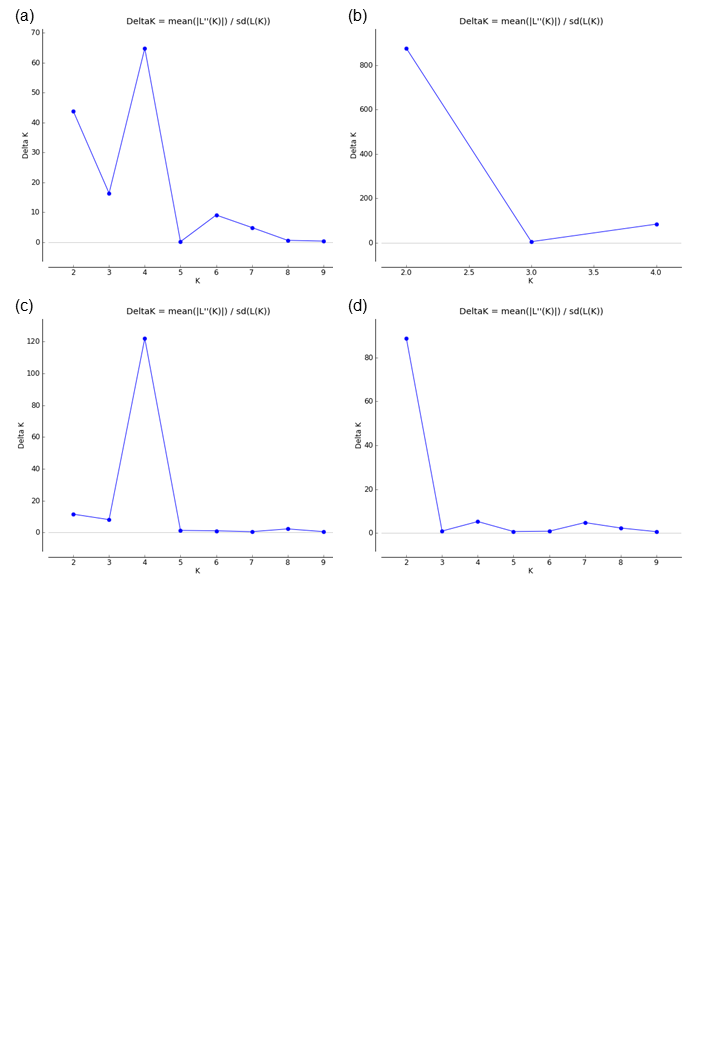

Supplement: Supplementary file 1 — Supplementary Information [file 41598_2018_38373_MOESM1_ESM.docx]
